# Effect of supplementation of *Aloe vera* extracts in cold storage media and cryopreservation of domestic cat epididymal spermatozoa

**DOI:** 10.21451/1984-3143-AR2019-0067

**Published:** 2020-01-30

**Authors:** Brenna de Sousa Barbosa, Fernanda Araujo dos Santos, Luãn Barbalho do Macêdo, Roberta Gonçalves Izzo, Denilsa Pires Fernandes, Érika Almeida Praxedes, Alexandre Rodrigues Silva, Marcelo Barbosa Bezerra

**Affiliations:** 1 Universidade Federal Rural do Semi-Árido – UFERSA – Mossoró, RN, Brasil

**Keywords:** freezing, tomcat, sperm, Aloe vera, ryoprotectant

## Abstract

This study evaluated the effect of the extract of *Aloe vera* at concentrations of 10% and 20% on the cryopreservation of sperm from the epididymis of domestic cats. Epididymal spermatozoa were recovered using the flotation technique and used in the treatments: control (TRIS-egg yolk at 20%), T10% (TRIS plus 10% of *A. vera* extract), and T20% (TRIS plus 20% of *A. vera* extract). The spermatozoa were subjected to 4ºC for 60 minutes, followed by 20 minutes in nitrogen vapors, and stored in a cryogenic cylinder. The samples were thawed at 37°C for 30 seconds. The sperm motility decreased (*P*<0.05) after thawing in the three treatments. Only the spermatozoa in the control treatment maintained post-thawing vigor. The viability of spermatozoa decreased in the treatments with *A. vera* (*P*<0.05). According to the hypoosmotic test, all treatments maintained the sperm membrane functionality (*P*>0.05) during freezing; however, after thawing, it decreased (*P*<0.05) in the T10% and T20% treatments. The morphology and chromatin condensation of spermatozoa did not differ, regardless of the treatments and time of evaluation (*P*>0.05). The effect of the crude *A. vera* extract was not satisfactory on the cryopreservation of epididymal spermatozoa of domestic cats after thawing; although the motility of spermatozoa was similar to that found with the use of egg yolk, and it presented maintenance of the chromatin integrity. However, it is necessary to understand the action of the substances present in *A. vera* with the feline spermatozoa, well as the standardization and adjustment of physicochemical characteristics aiming at the future application of the vegetal extract.

## Introduction

Cryopreservation of epididymal spermatozoa of domestic cats is a wide-range research area because of the need to create a gene bank for endangered felines. Moreover, it allows the obtaining of bred animals of high commercial value by recovering spermatozoa after castration or necropsy, and subsequent use in breeding programs (Chatdarong, 2017; Brusentsev et al., 2018).

However, the post-thawing quality of the spermatozoa is significantly reduced, with motility loss of 40%-60% (Kunkitti et al., 2017). According to some researches on handling and preserving spermatozoa, feline spermatozoa have peculiar morphological and physiological characteristics, such as teratozoospermia, phenomenon in which ≥ 60% of the gametes of an sperm sample have abnormalities (Filliers et al., 2010; Macente et al., 2012; Martins and Justino, 2015). Teratozoospermic spermatozoa are more susceptible to cryogenic damages, when compared with the normozoospermic ones, often making not possible their cryopreservation (Pukazhenthi et al., 1999, 2000; Kalthur et al., 2008). Teratozoospermia was found in twenty-eight species of felines, including domestic cat, which present individuals with normozoospermia and teratozoospermia (Müller et al., 2012).

Cryopreservation protocols for domestic cats describe glycerol (4% or 5%) and egg yolk (10-20%) associated with the diluents TRIS (tris(hydroxymethyl)aminomethane) or TEST (N-tris-hydroxymethyl-methyl- 2-aminomethane) as cryoprotectants (Buranaamnuay, 2017). Although egg yolk is an excellent extracellular cryoprotectant, there are controversies about its use; it has been reported as having substances that hinder cellular respiration, affecting the electron transport system in the mitochondria of the spermatozoa. Moreover, egg yolk is a potential propagator of pathogenic microorganisms, thus, the exchange of the cryopreserved biological material with this substance is restricted (Bousseau et al., 1998; Souza et al., 2014).

Therefore, the ability of several substances of plant origin to protect spermatozoa on cryopreservation are being assessed. The use of *Aloe vera* for this purpose is a possibility; it has similar substances to conventional cryoprotectants in its composition (Souza et al., 2014). The cryoprotective action of *A. vera* has been identified in refrigeration of semen of goats (Aguiar et al., 2012), cattle (Vargas, 2012), dogs (Melo et al., 2014) and peccaries (Souza et al., 2016). The antioxidant, antimicrobial and cellular regeneration properties of *A. vera* can be used in the production of more favorable means for spermatozoa survival during cryopreservation (Estakhr and Javdan, 2011; Nejatzadeh-Barandozi, 2013; Rozkot et al., 2013; Freitas et al., 2014).

In this context, the objective of this work was to evaluate the cryoprotective effect of two concentrations of *A. vera* extract on the cryopreservation of epididymal spermatozoa of domestic cats.

## Methods

### Preparation of the *A. vera* extract

The plant *Aloe vera* were collected in Mossoró RN, located in the northeasr of Brazil, Brazil, between 6:15 a.m. and 6:30 a.m. The crude extract of the plant was obtained by removing the outer layer of the cataphile and extraction the parenchyma of the leaf, which has a mucilaginous colorless aspect. This material was sieved and placed in a sterile glass container in which diluent solutions were added (Aguiar et al., 2012).

### Experimental design

The local Animal Ethics Committee approved all the procedures (protocol number 23091.001390/2018-11). Twelve adult male cats, aged 1.5 to 4 years, underwent routine castration at the institution's veterinary hospital, were included in this study. The pair of testicular-epididymal complexes were placed in 0.9% saline solution at temperature 37 ºC and transported to the laboratory within 1 hours.

Spermatozoa recovered (fresh) from each epididymal pair (n = 12) were evaluated for sperm concentration, total motility, vigor, viability, functional membrane integrity, morphology and chromatin integrity. The sample was divided into three aliquots, diluted in the different treatments: Control, T0% and T20%, equilibrated by cold storage for 1 hour, and cryopreserved. Beforer to cryopreservation (cooling), the sperm quality of the epididymis was repeatedly assessed, except for analysis of chromatin condensation. The thawing of samples sperm was performed one week after freezing; these have been repeatedly evaluated (post-thawing).

### Epididymal sperm collection, freezing, and thawing

Epididymal sperm were recovered by the slicing technique. The tail portion of the epididymis pair was sectioned and incubated in contact with 1 mL of the TRIS buffer (Tris-hidroximetil-aminometano plus fructose and citric acid monohydrate) for five minutes at 37°C (Silva and Mota-Filho, 2012). Thereafter, the tissue fragments were removed and the recovered sperm transferred to a 2 ml eppendorf tube (preheated to 37 °C), which was subjected to centrifugation of 500g for 6 minutes. Formed *pellet* was resuspended in TRIS (Cocchia et al., 2010).

The TRIS buffer containing sperm was equally fractionated into three eppendorfs tubes which were intended for the treatments: Control (TRIS plus 20% egg yolk), T10% (TRIS plus 10% *A. vera*) and T20% (TRIS plus 20% *A. vera*). The diluent of each treatment was added to an initial concentration of 100 x 10^6^ sptz/mL. Then the samples were submitted to the equilibrium curve at 4°C for 60 minutes. Subsequently, diluents of similar composition plus 8% glycerol (final concentration of 4% glycerol) was added to the samples (final concentration in 50 x 10^6^ sptz/mL). Sperm samples were kept for a further 5 minutes at 4°C. The spermatozoa were then packed in 250 μL plastic straws, exposed to nitrogen vapors (distance of 6 cm from the surface of nitrogen) for 20 minutes and stored in a cryogenic bottle (Cocchia et al., 2010).

The thawing of straws was proceeded at 37°C for 30 seconds (Cocchia et al., 2010). The spermatozoa were maintained in microtubes at 37ºC, and immediately submitted to sperm evaluation.

### Spermatozoa evaluation

The total motility (percentage of moving cells) and vigor (quality of progressive spermatozoa movement on a scale of 0 to 5) was evaluate using 5 μL of sperm sample placed on a preheated slide (37ºC),with observations of two to three fields different from the sample under magnification of 100X under optical microscope (CBRA, 2013).

The plasma membrane functionality was evaluated by the hypoosmotic test. Therefore, 5 μL aliquot was incubated in a defined osmolarity medium (150mOsm /L) for 45-60 minutes at 37 °C and subsequently evaluated by an optical microscope (400X), where 100 spermatozoa were counted randomly, between rolled tails or not. The percentage of coil-tailed spermatozoa were considered as membrane functional integrity (Emerenciano et al., 2013).

The viability test consisted of the smeared spermatozoa with the Bromophenol blue dye followed by counting 100 cells using an optical microscope (400x), which were classified as non-viable (blue staining) or viable (unstaining) (Lima et al., 2016).

The morphology was evaluated by staining the spermatozoa with a 1.5% Rose Bengal solution and counting 200 random cells, quantifying them as normal and abnormal, using an optical microscope (1000x) (Emerenciano et al., 2013). In the counting of abnormal sperm were considered the head defects (double, macro or microcephaly, abnormal shape, detached head); intermediate piece defects (folded, thickened, segmented, fibrillary); tail defects (coiled, heavily rolled, folded, double, with inserted abaxial or paraaxial) and presence of cytoplasmic droplet (proximal or distal) (Arruda et al., 2011).

The chromatin condensation test consisted of smearing 10 μL of the sample on a glass slide fixed according to the protocol described by Kamimura et al. (2010). The slide was immersed for 1 minute in an ethanol-acetic acid solution (3:1), transferred to a 70% ethanol solution, where it remained for 3 minutes, and then to 4M hydrochloric acid solution for 25 minutes. The slide was then washed with distilled water and left to dry naturally. After, 5 μL of toluidine blue dye was added to the dry slide for the 500 sperm counts and classify them as little stained (or light blue) for condensed chromatin, and intensely stained (violet or dark blue) for decondensed chromatin.

### Statistical analysis

The data were analyzed in the statistical programs BioEstat 5.3 and Statistica 8.0, and expressed as mean and standard error. Normality and homoscedasticity tests were performed Shapiro-Wilk and Kolmogorov-Smirnov tests, respectively. The Kruskal-Wallis test was chosen, followed by the Dunn test to compare the total motility, viability, membrane functionality, morphology and analysis of chromatin condensation of the treatments control, T10% and T20%. Sperm vigor was evaluated by the Mann-Whitney test. All results were considered significant when *P*<0.05.

## Results

The results of the evaluation of the diluted epididymal spermatozoa subjected to freezing-thawing in the different treatments are shown in Table 1.

**Table 1:** Characteristics of sperm samples from domestic cat epididymis by groups (fresh sample, cooled sample, and thawed sample) and treatments (Control, T10% and T20%) for the variables motility, vigor, membrane functional integrity, viability, morphology, and chromatin condensation.

**Treatment**		**Motility (%)**	**Vigor (0-5)**	**Membrane functional integrity (%)**	**Normal morphology (%)**	**Viability (%)**	**Chromatin condensed (%)**
Fresh		72.28 ± 2.90^a^	3.67 ± 0.17^a^	73.89 ± 4.13^a^	41.89 ± 3.83^a^	66.30 ± 1.39^a^	99.80 ± 0.18
Cooled	Control	59.44 ± 2.93^aA^	3.22 ± 0.15^aA^	64.44 ± 3.39^aA^	40.17± 1.80^aA^	54.70 ± 3.19^aA^	-
	T10%	48.33 ± 5.95^aA^	2.33 ± 0.37^bA^	49.78 ± 5.00^aA^	38.28 ± 3.21^aA^	41.80 ± 3.44^bB^	-
	T20%	48.33 ± 6.77^aA^	2.56 ± 0.34^bA^	55.78 ± 1.78^aA^	37.44 ± 1.66^aA^	43.76 ± 3.97^bB^	-
Post-thawing	Control	36.33 ± 3.71^bA^	3.22 ± 0.15^aA^	52.89 ± 2.16^aA^	40.67 ± 2.31^aA^	36.40 ± 2.63^aA^	99.76 ± 0.24
	T10%	12.78 ± 2.64^bB^	1.78 ± 0.40^bB^	35.56 ± 1.07^bB^	35.89 ± 2.56^aA^	17.44 ± 2.76^bB^	99.84 ± 0.18
	T20%	22.78 ± 5.34^bAB^	2.11 ± 0.45^bB^	41.00 ± 3.61^bB^	35.83 ± 2.60^aA^	24.11 ± 1.89^bB^	99.98 ± 0.11

a, b, c Different lowercase letters in the columns indicate differences between fresh, cooled, and post-thawing sperm samples (*P*<0.05);^A, B, C^ Different uppercase letters in the columns indicate differences between treatments (Control, T10% and T20%) (*P*<0.05).

The total motility of the fresh (72.28 ± 2.90%) and cooled spermatozoa was similar in the control (59.44 ± 2.93%), T10% (48.33 ± 5.95%) and T20% (48.33 ± 6.77%) treatments; and no differences (*P*>0.05) were found between treatments in the cooling evaluation. The thawed samples showed a significant reduction in total motility compared to fresh samples (*P*<0.05), with values of 36.33 ± 3.71% for control, 12.78 ± 2.64% for T10% and 22.78 ± 5.34% for T20%.

The total motility in T10% was lower than in the control (*P*<0.05) after thawing. Though, the T20% treatment showed similar motility to both control and T10% (*P*>0.05).

The sperm vigor of fresh and post-thawed samples was similar in control (*P*>0.05), different from that observed for treatments using *A. vera* as a cryoprotectant, irrespective of the concentration (*P*<0.05). The sperm vigor of the post-thawing treatments was different (*P*<0.05), with values of 1.78 ± 0.40 for T10%, 2.11 ± 0.45 for T20%, and 3.22 ± 0.15 for the control.

According to the hypoosmotic test, all treatments (control, T10%, T20%) maintained membrane functionality during cooling (*P*>0.05). However, the values of T10% and T20% decreased (*P*<0.05) at post-thawing to 35.56 ± 1.07% and 41.00 ± 3.61%, respectively. These values were different (*P*<0.05) from those of the control (52.89 ± 2.16%).

The sperm viability decreased (*P*<0.05) in T10% and T20% at cooling and post-thawing in relation to fresh sample; while the control maintained similar percentage of viable cells throughout freeze-thaw (*P*>0.05).

The morphology presented no differences (*P*>0.05) between treatments throughout the cryopreservation. The same result was found for chromatin condensation (*P*>0.05).

## Discussion

Evaluating sperm parameters is fundamental to understand the extent of the damage caused to the spermatozoa during cryopreservation, as well as analyzing protocols and choosing diluents, and cryoprotectants to better protect the cell mechanisms (Cheuquemán et al., 2018). The present study showed that both external cryoprotectants (egg yolk and *A. vera*) maintained the sperm motility during cooling, independent of the concentration of *A. vera* used in the diluent; however, the sperm motility after thawing was similar between the diluents composed of egg yolk or *A. vera*, only when the plant extract was used in the same concentration of the usual extracellular cryoprotectant, indicating that a possible cryoprotective effect on feline sperm requires concentration above 10% of *A. vera*. Some authors hypothesize that spermatozoa diluted with *A. vera* require a longer time for adaptation due the mucilaginous characteristic that the plant extract presents (Melo et al., 2014); maybe incubation of feline spermatozoa with extender containing *A. vera* for more than 1 hour time would significantly benefit sperm motility. We also hypothesized that the use of intermediate concentrations of *A. vera* extract (greater than 10% but below 20%) may have a better response to sperm cell because this plant extract has high viscosity and can restrict sperm movement of viable cells (Farias, 2017).

In addition, similarities between the T10% and T20% treatments observed in the total motility parameter can be explained by the physical-chemical characteristics of the *A. vera* gel and its mechanisms for sperm protection, which are due to the presence of polysaccharides and saponins. The polysaccharides, rich in xylose, glucose, and galactose, promote the reorganization and stabilization of the protein components of plasma membrane, through the formation of hydrogen bonds, preventing protein denaturation during cryopreservation; these sugars also act in the dehydration of the cell, reducing the formation of intracellular ice crystals. While the saponins act as surfactants, protecting the plasma membrane by preventing the formation of micelles (Aguiar et al., 2012; Vargas, 2012; Di Domenico et al., 2015).

Regarding the cell viability, egg yolk presented better results as external cryoprotectant than *A. vera* in both 10% and 20% concentrations. This result is explained by the egg yolk mechanism of action, which promotes stabilization of the plasma membranes and replaces the phospholipids that compose them (Sieme et al., 2016); whereas the interaction of *A. vera* polysaccharides with membrane components are little effective in protecting them against cryoinjuries.

The maintenance of viability is fundamental for sperm membranes remain intact and the gamete interact with the oocyte at fertilization. Cryopreservation promotes phospholipid translocation phenomena between the external and internal side of the lipid bilayer and premature acrosome reaction, decreasing the viability of cryopreserved spermatozoa when compared to fresh spermatozoa (Silva and Guerra, 2011). The viability values found were consistent with those described in the literature for fresh epididymal spermatozoa (41.7% to 74.3%), whereas for cryopreserved spermatozoa (33.5% to 63.9%), only the control (35.56 ± 2.77%) was within the reported range (Hermansson and Axnér, 2007; Chatdarong et al., 2010; Cocchia et al., 2010; Emerenciano et al., 2013; Kunkitti et al., 2016).

The results of the viability test may be associated with the hypoosmotic test and thus indicate the fertility of a sperm sample (Bittencourt et al., 2006). The hypoosmotic test allows to distinguish which cells have functional membrane, based on the liquid transport dynamics of the intact plasma membranes in media with different solute concentrations (Santos et al., 2016). The hypoosmotic test values obtained with the use of *A. vera* in cryopreservation of feline spermatozoa are consistent with the low values of viability found. However, all functional membrane integrity results found after thawing agree with the reported range (37%-90%) for epididymal spermatozoa in the domestic feline. This large variation in the percentage of functional membrane cells in the sample is a limiting factor of the development of an cryopreservation protocol in the species (Emerenciano et al., 2013). Therefore, the understanding of cryoprotective synergism with the sperm cell should be investigated in order to obtain a diluent capable of maintaining the stability and integrity of the plasma membrane and consequently the osmoregulation mechanisms of the cell throughout the cryopreservation (Figueroa et al., 2016).

Morphological analysis is important to determine the sperm sample quality and, consequently, the fertility of the male. Positive correlations between percentage of normal spermatozoa, sensitivity to refrigeration, and *in vitro* fertility rate was confirmed for feline species (Howard et al., 1993; Pukazhenthi et al., 1999, 2000; Penfold et al., 2003 **;** Villaverde et al., 2008; García-Vázquez et al., 2016). Sperm of felines frequently present high rate of abnormality, with average of 44% of normal spermatozoa (Axnér and Forsberg, 2007). The main abnormalities found in this study were presence of coiled and broken tail, and folded intermediate region. No divergence was found between the treatments, or between the fresh, cooled, and thawed results, thus confirming that the use of *A. vera* as an extracellular cryoprotectant does not have negative effects on the morphology of spermatozoa.

The use of *A. vera* presented satisfactory results regarding chromatin integrity after thawing. This is probably due to the excellent stability of feline sperm chromatin and its cryostress tolerance, as has been reported (Kunkitti et al., 2016). The chromatin compaction level is important for the preservation of the genetic material and transmission of the genetic characteristics to the offspring. Much of the mature spermatozoa has compacted chromatin; this condensation begins in the testes and continues during the passage of the spermatozoid through the epididymis, where the replacement of the histones by the protamines occurs, which will be stabilized by the formation of disulfide bridges (Sringnam et al., 2011; Nava-Trujillo et al., 2012). However, cryopreservation can generate damage to the nuclear and/or mitochondrial DNA of the sperm cell; thus affect the integrity or expression of some proteins, enzymatic activity and the phosphorylation profile in the gametes (Figueroa et al., 2016). The status of DNA integrity can be evaluated by several methods, such as the comet assay, tunel assay and toluidine blue staining. The evaluation by toluidine blue staining, used in the work, is a simple, low cost and validated technique for several species, including domestic cat (Rui et al., 2017). The results obtained from this analysis allow us to envisage the future application of cryopreserved epididymal spermatozoa in intracytoplasmic injection biotechnology (ICSI), since more than 90% of the cells had condensed chromatin. However, specific techniques of DNA integrity analysis, such as quantitative polymerase chain reaction (qPCR), must be performed in the future to ratify the results found in this research.

Another finding of the study was the notorious observation of the less dirt aspect of the cryopreserved sperm samples with *A. vera,* different from those with egg yolk, that may form lumps during the cryopreservation steps and hinder the subsequent use of the spermatozoa. Moreover, the antimicrobial activity of *A. vera* already reported in other studies can be explored in the conservation of spermatozoa of domestic cat and of other wild feline (Nejatzadeh-Barandozi, 2013; Farias, 2017).

## Conclusion

The effect of the crude *A. vera* extract was not satisfactory on the cryopreservation of epididymal spermatozoa of domestic cats after thawing, although the motility of spermatozoa was similar to that found with the use of egg yolk, and it presented maintenance of the chromatin integrity.

The use of *Aloe vera* in the preservation of feline spermatozoa is incipient and the results can be optimized with a better understanding of the mechanisms of interaction between the substances present in the plant extract and sperm cells, even as it is necessary to establish a standard protocol for the use of *A. vera* extract in cryopreservation of spermatozoa.

Standardization and adjustment of physicochemical characteristics of this vegetal extract can favor the survival of the cryopreserved spermatozoa and, thus, make possible the application of this natural product as an alternative cryoprotectant diluent for spermatozoa of domestic felines, and in the future, for wild felines.

## List of abbreviations used

*Aloe vera* – *A. vera.*

Tris- hydroxymethyl(aminomethane) – TRIS.

N-tris-hydroxymethyl-methyl- 2-aminomethane **–** TEST.

TRIS Diluent plus 10% *Aloe vera* – T10%.

TRIS Diluent plus 20% *Aloe vera* – T20%.
